# Involvement of the Serine Protease Inhibitor, SERPINE2, and the Urokinase Plasminogen Activator in Cumulus Expansion and Oocyte Maturation

**DOI:** 10.1371/journal.pone.0074602

**Published:** 2013-08-30

**Authors:** Chung-Hao Lu, Robert Kuo-Kuang Lee, Yuh-Ming Hwu, Ming-Huei Lin, Ling-Yu Yeh, Ying-Jie Chen, Shau-Ping Lin, Sheng-Hsiang Li

**Affiliations:** 1 Department of Medical Research, Mackay Memorial Hospital, Tamshui, New Taipei City, Taiwan; 2 Institute of Biotechnology, College of Bio-Resources and Agriculture, National Taiwan University, Taipei, Taiwan; 3 Department of Obstetrics and Gynecology, Mackay Memorial Hospital, Taipei, Taiwan; 4 Department of Obstetrics and Gynecology, Taipei Medical University, Taipei, Taiwan; 5 Mackay Medicine, Nursing and Management College, Taipei, Taiwan; 6 Genomics Research Center and Agricultural Biotechnology Research Center, Academia Sinica, Taipei, Taiwan; Baylor College of Medicine, United States of America

## Abstract

The serpin peptidase inhibitor, clade E, member 2 (SERPINE2) inhibits urokinase-type plasminogen activator (PLAU) and tissue-type plasminogen activator. Higher *SERPINE2* expression levels were detected in cumulus cells of human immature oocytes than in those of mature oocytes. The objective of this study was to evaluate whether high SERPINE2 levels in cumulus cells are associated with oocyte immaturity. Using the mouse cumulus–oocyte complex as an experimental model, the effects of elimination and overexpression of SERPINE2 in cumulus cells on cumulus expansion and oocyte maturation were assayed by *in vitro* maturation. *Serpine2* and *PLAU* transcripts were the most highly expressed serpins and plasminogen activators, respectively. Their expression was coordinately regulated in cumulus cells during gonadotropin-induced oocyte maturation. Silencing of *Serpine2* expression using small interfering RNAs or blockage of SERPINE2 protein using a specific antibody had no effect on oocyte maturation. However, overexpression of *Serpine2* or exogenous supplementation with high levels of SERPINE2 impaired cumulus expansion and oocyte maturation, probably by decreasing hyaluronan synthase 2 (*Has2*) and versican (*Vcan*) mRNA expression. Amiloride, a specific PLAU inhibitor, also suppressed these processes. PLAU supplementation of the oocyte *in vitro* maturation medium caused earlier and more extensive expansion of cumulus cells and oocyte maturation that may be mediated by increased *Has2* mRNA expression. However, these effects were neutralized by coincubation with SERPINE2 or amiloride and PLAU. In conclusion, SERPINE2 and PLAU are involved in cumulus expansion and oocyte maturation. High SERPINE2 levels impair these processes, probably by decreasing cumulus matrix gene expression as well as reducing cumulus hyaluronan contents and inhibiting PLAU activity. These findings may explain why cumulus cells surrounding immature human oocytes express high SERPINE2 levels.

## Introduction

The structural integrity of the cumulus cell extracellular matrix (ECM) is essential for oocyte maturation. Several cumulus proteins linked to ECM hyaluronan, e.g., heavy chain of inter-alpha-trypsin inhibitor (ITIH) [1], pentraxin-3 (PTX3) [2], [3], tumor necrosis factor alpha-induced protein 6 (TNFAIP6) [4], and versican (VCAN) [5], are required for regulating cumulus integrity, thus ensuring cumulus expansion and oocyte maturation [2], [6], [7]. Cumulus expansion involves hyaluronan accumulation in the intercellular spaces of cumulus cells, and its induction by gonadotropins is crucial for oocyte maturation [8]. Oocyte-secreted molecules, e.g., growth differentiation factor 9 and bone morphogenetic protein 15, also affect cumulus expansion [9], [10]. Thus, bidirectional intercellular communication between oocytes and their surrounding cumulus cells is important for the development of an egg that is competent to undergo fertilization and embryogenesis [8], [11], [12].

Plasminogen activators (PAs) are associated with many reproductive processes, e.g., ovulation [13]–[17], embryonic development [18], and embryo implantation [19], and pathological processes, e.g., tumor invasion [20]. PAs are involved in tissue remodeling by converting abundant extracellular plasminogen into active protease plasmin, which degrades almost all matrix proteins [20]. Two PA types, tissue (PLAT) and urokinase (PLAU), and four types of serine protease inhibitors (serpins), including SERPINA5, SERPINB2, SERPINE1, and SERPINE2, constitute the PA system [20]. Understanding how serpins modulate PLAT/PLAU proteolytic activities is considerably important in developing therapeutic strategies for PA-involved tissue remodeling.

SERPINE2 has broad-spectrum protease inhibitory activities toward trypsin, thrombin (F2), plasmin, PLAU [21], and prostasin (PRSS8) [22]. It is extensively expressed in reproductive tissues, e.g., the placenta [23], [24], uterus [24], [25], ovary [26], [27], and seminal vesicles [28]. In addition to its protease inhibitor role, SERPINE2 functions as a sperm decapacitation factor [29]. During ovulation, SERPINE2 and PLAU expression is coordinated in mice [27], whereas SERPINE1 and PLAT expression is coordinated in monkeys and rats [15], [30], [31]. This indicates that the PA system has species-specific expression patterns in the ovary.

PA expression levels upregulated in cumulus cells just before ovulation [13] suggests involvement in follicle wall rupture during ovulation [13]–[17]. However, Liu et al showed that plasmin, the best known target of PA, was not critical for ovulation [32]. PAs and their cognate serpin inhibitors have been detected in cumulus cells [27]; nevertheless, their involvement in oocyte maturation during pre-ovulation needs clarification. Higher *SERPINE2* expression levels were detected in cumulus cells of human immature oocytes than in those of mature oocytes [33]. Therefore, we here assumed that high SERPINE2 levels were correlated with oocyte immaturity. To verify this, we used mouse cumulus–oocyte complexes (COCs) as a model for evaluating the association of SERPINE2 levels with cumulus expansion and subsequent oocyte maturation.

## Materials and Methods

### Ethics statement

This study was approved by the Mackay Memorial Hospital Institutional Review Board (reference number 09MMHIS024) with written consent for the use of human cumulus cells. Written consent for the use of cumulus cells was obtained from 20 patients undergoing intracytoplasmic sperm injection treatment. All animals contributed to this study were maintained in the Animal Center at the Department of Medical Research, Mackay Memorial Hospital. The animal use protocol has been reviewed and approved by the Mackay Memorial Hospital Institutional Animal Care and Use Committee with an approval number MMH-A-S-100-45. All efforts were made to minimize suffering.

### Collection of human cumulus cells

Patients undergoing classical *in vitro* fertilization treatments at the Center of Reproductive Medicine, Mackay Memorial Hospital, Taiwan received controlled ovarian hyperstimulation by application of the gonadotropin-releasing hormone antagonist protocol. COCs from follicles >14 mm were collected using transvaginal ultrasound and a 16-gauge needle and were exposed to 80 IU hyaluronidase in Quinn's Advantage Fertilization medium (Sage BioPharma, Bedminster, NJ) for 20 s at 37°C to dissolve hyaluronan. Of the 46 COCs, 26 and 20 had mature and immature oocytes, respectively. The cumulus cells were individually separated from the COCs under an Olympus SZX7 stereomicroscope (Tokyo, Japan). They were mixed with 20 µl of extraction buffer from the Arcturus PicoPure RNA Isolation Kit (Applied Biosystems, Foster City, CA) for total RNA isolation and stored at −80°C until use. Cumulus cells individually collected from 10 other COCs were fixed on slides using 4% (v/v) paraformaldehyde for immunohistochemical staining.

### Collection of mouse cumulus cells

The mice (age, 21–24 days) were injected with 5 IU of pregnant mare serum gonadotropin (PMSG; Sigma-Aldrich, St. Louis, MO) and sacrificed by cervical dislocation after 46 h. The ovaries were removed and briefly rinsed with PBS. COCs were isolated by puncturing antral follicles with a 30-gauge needle under an Olympus SZX7 stereomicroscope. To study the effect of luteinizing hormone on *Serpine2* and *Plau* expression in cumulus cells during oocyte maturation, PMSG-primed mice were injected with 5 IU of human chorionic gonadotropin (hCG; Sigma-Aldrich). Ovaries were removed 3, 6, and 9 h after hCG treatment. COCs were isolated by puncturing antral follicles as described above. For post-ovulation COCs, the ovaries were removed 12 h after hCG injection, and the COCs were collected by flushing the oviducts with PBS. All COCs were treated with 150 IU hyaluronidase in PBS for 3 min at 37°C, the oocytes were removed, and cumulus cells were collected by centrifugation at 1000 ×*g* for 3 min at room temperature.

### Quantitative real-time RT-PCR (qRT-PCR)

Total RNA of cumulus cells was extracted using the Arcturus PicoPure RNA Isolation Kit and directly reverse transcribed into a 50 µl first-strand cDNA pool using a High Capacity cDNA Archive Kit (Applied Biosystems) according to the manufacturer's instructions. qRT-PCR was performed using primers (Table S1) [24]. The housekeeping genes, human ribosomal protein L19 and mouse hypoxanthine guanine phosphoribosyltransferase gene, were used as internal loading controls to normalize relative gene expression levels. PCR amplification efficiency for each tested gene was examined to ensure that it was equivalent to that of the housekeeping gene examined in a cDNA dilution series. PCR was performed in a total volume of 20 µl, containing 50 ng of tissue cDNA, 250 nM each of the forward and reverse primers, and 10 µl of 2× SYBR Green Master Mix (Applied Biosystems). All reactions were performed in triplicate and run on an ABI/PRISM 7500 Fast Sequence Detection System (Applied Biosystems) under the following conditions: 95°C for 20 s, and then 40 cycles at 95°C for 3 s and 60°C for 30 s. The threshold cycle (Ct) was defined as the fractional cycle number at which the reporter fluorescence, i.e., the number of amplified copies, reached a fixed threshold. Melting curve analysis was performed to verify that only a single product had formed in the reaction. The identity of the PCR products was confirmed by DNA sequencing. Relative quantification of mRNA expression was calculated by the 2^−ΔΔCt^ method [34].

### SERPINE2 proteins and anti-SERPINE2 antiserum

SERPINE2 proteins and anti-SERPINE2 antiserum were prepared [29]. To prepare control antiserum, anti-SERPINE2 antiserum was adsorbed onto SERPINE2-conjugated beads for removing the specific anti-SERPINE2 antibody [29].

### SERPINE2 and PLAU immunolocalization and hyaluronan status on COCs

COCs were transferred onto slides, air dried, and fixed in 4% paraformaldehyde for 15 min. The slides were incubated in blocking solution [10% (v/v) normal goat serum in PBS] for 1 h at room temperature and then incubated with anti-SERPINE2 or control antiserum (1∶1000), with rabbit anti-PLAU antiserum (1∶100; Santa Cruz Biotechnology, Santa Cruz, CA), or with pre-immune rabbit serum (1∶500; Jackson ImmunoResearch, West Grove, PA) in blocking solution at 4°C for 16 h. To assess the hyaluronan status in cumulus cells, slides were incubated with biotinylated hyaluronic acid binding protein (HABP; 1∶200, Sigma-Aldrich, cat. no. H9910) in blocking solution at 4°C for 4 h. After washing three times in PBS with slight agitation for 5 min each, the slides were treated with fluorescein isothiocyanate-conjugated goat anti-rabbit IgG (1∶1000; Jackson ImmunoResearch) or with tetramethyl rhodamine isothiocyanate-conjugated goat anti-rabbit IgG (1∶1000; Jackson ImmunoResearch) in blocking solution for 1 h at room temperature or with streptavidin-conjugated Alexa Fluor 488 (1∶1000; Jackson ImmunoResearch) in blocking solution for 2 h at room temperature. The slides were washed again and then counterstained with 5 µg/ml Hoechst 33258. After three brief rinses with PBS, the slides were mounted in 100 µl of ProLong Gold antifade medium (Invitrogen Molecular Probes, Eugene, OR) and photographed using an epifluorescence microscope (Olympus BX 40) equipped with an Olympus DP-70 digital camera.

### *In vitro* maturation (IVM)

To assess the extent of cumulus cell expansion, COCs isolated from PMSG-primed ovaries that had even diameters of approximately 100 µm and contained a nucleus (germinal vesicle, GV) were cultured in IVM medium as described previously [35], [36] with some modifications. The IVM medium consisted of MEMα medium (Life Technologies, Grand Island, NY) supplemented with 10% fetal bovine serum (Sigma-Aldrich), 0.23 mM sodium pyruvate, 75 mU/ml follicle-stimulating hormone (FSH), 50 mg/l streptomycin, 60 mg/l penicillin, and 1 µg/l epidermal growth factor (EGF), pH 7.4. COCs were incubated in 150-µl microdrops of IVM medium supplemented with SERPINE2 (0.03, 0.06, or 0.12 mg/ml), anti-SERPINE2 antibody (1∶1000), amiloride (300 µM), or PLAU (20 U; Millipore, Billerica, MA) and overlaid with mineral oil for approximately 16–20 h in a humidified 5% CO_2_ atmosphere at 37°C. For control experiments, COCs were incubated in IVM medium without supplementation. After IVM, the diameters of expanded cumulus cells were scored. Next, the COCs were treated with 150 IU hyaluronidase in IVM medium for 3 min at 37°C, and cumulus cells were removed by repeated pipetting. The morphology of oocyte nuclei was observed, and the oocytes were classified as immature [GV or metaphase I (MI) stage] or mature (MII stage, with the extrusion of the first polar body). Oocyte maturation rate was determined after 16 h of culture by counting the number of MII oocytes among the total oocytes used in an assay.

### Treatment of COCs with small interfering RNA (siRNA)

siRNA against mouse *Serpine2* (catalog #20720-Serpine2; Dharmacon, Thermo Fisher Scientific, Lafayette, CO) and a non-targeting negative control siRNA (catalog #D-001206-05; Dharmacon, Thermo Fisher Scientific) dissolved in Accell siRNA delivery media were used according to the manufacturer's instructions. COCs isolated from PMSG-primed ovaries were incubated with 1, 2, or 3 µM siRNAs for 24 h in 150 µl MEMα medium supplemented with 10 µM milrinone (a phosphodiesterase inhibitor, Sigma-Aldrich, cat. no. M4659), 50 mg/l streptomycin, 60 mg/l penicillin, 0.23 mM pyruvate, and 3 mg/ml bovine serum albumin (Sigma-Aldrich). The optimal concentration for both siRNAs was 3 µM. After 24 h incubation, the COCs were transferred to IVM medium and cultured in a humidified 5% CO_2_ atmosphere at 37°C for 16 h. Cumulus expansion and oocyte maturation were then assessed as described above. *Serpine2* mRNA levels in cumulus cells were examined by qRT-PCR. To analyze whether SERPINE2 protein was knocked down, COCs were transferred onto slides and examined by immunohistochemistry as described above.

### Construction of the mouse *Serpine2* expression vector

The DNA fragment of the pIRES2-DsRed2 vector (Clontech Laboratories, Mountain View, CA) containing the multiple cloning site (MCS) and the red fluorescence protein coding region (DsRed2) was amplified by PCR using primer pairs bearing *EcoR*I sites (forward primer 5′-TTCGAATTCTGCAGTCGACGGTACC-3′, reverse primer 5′-TTTGAATTCATCTAGAGTCGCGGCCGC-3′; Figure S1a). Thirty-five PCR cycles were performed under the following conditions: denaturation at 95°C for 30 s, annealing at 55°C for 30 s, and extension at 72°C for 1 min. The PCR product was verified by agarose gel electrophoresis and DNA sequencing and ligated into an *EcoR*I-digested pCX-EGFP vector (Addgene, Cambridge, MA) to form the pCX-DsRed2 intermediate vector (Figure S1b and c).

Since *Serpine2* is predominantly expressed in mouse seminal vesicles [28], total RNA was extracted from that tissue using an RNeasy Mini Kit (Qiagen, Valencia, CA) and reverse transcribed into cDNA with a High-Capacity cDNA Archive Kit (Applied Biosystems) according to the manufacturer’s instructions. The 1220-bp full-length mouse *Serpine2* cDNA (NCBI Reference Sequence NM_009255.4) was amplified by RT-PCR from the cDNA pool using a *Serpine2* primer pair (forward primer 5′-GAAGGAACCATGAATTGGC-3′, reverse primer 5′-TTCCTTTGTCTGTCCTTCAGG-3′). Thirty-five cycles of PCR were performed under the following conditions: denaturation at 95°C for 30 s, annealing at 55°C for 30 s, and extension at 72°C for 1 min. The PCR product was verified by agarose gel electrophoresis and DNA sequencing and cloned into the pGEM-T Easy vector (Promega, Madison, WI) by TA cloning.

The full-length *Serpine2* cDNA was excised with *Xma*I and cloned into MCS of the pCX-DsRed2 vector to create the *Serpine2* expression vector pCX-Serpine2-DsRed2 (Figure S1d). The construct was sequenced to verify the sequence and orientation of the reading frame. This construct enabled the simultaneously translation of both SERPINE2 and DsRed2 for monitoring SERPINE2 protein expression by red fluorescence.

### *Serpine2* overexpression in COCs

COCs isolated from PMSG-primed ovaries were transfected with 500 ng of the *Serpine2* expression vector pCX-Serpine2-DsRed2 or the vehicle vector pCX-DsRed2 using PolyJet DNA In Vitro Transfection Reagent (SignaGen Laboratories, Gaithersburg, MD) in 150 µl of MEMα medium supplemented with 10 µM milrinone (as mentioned above) but without FSH and EGF for 12 h. The COCs were washed three times using IVM medium, transferred to fresh medium, and cultured in a humidified 5% CO_2_ atmosphere at 37°C for 16 h. Cumulus expansion and oocyte maturation were assessed as described above. *Serpine2* mRNA levels in cumulus cells were examined by qRT-PCR.

### Statistical analysis

Data are presented as means ± SD. Differences were analyzed by one-way analysis of variance followed by the Bonferroni *post hoc* test using GraphPad Prism 5.0 (GraphPad Software, San Diego, CA). *P*<0.05 was considered significant.

## Results

### SERPINE2 was highly expressed in cumulus cells of immature human oocytes

**Figure 1:**
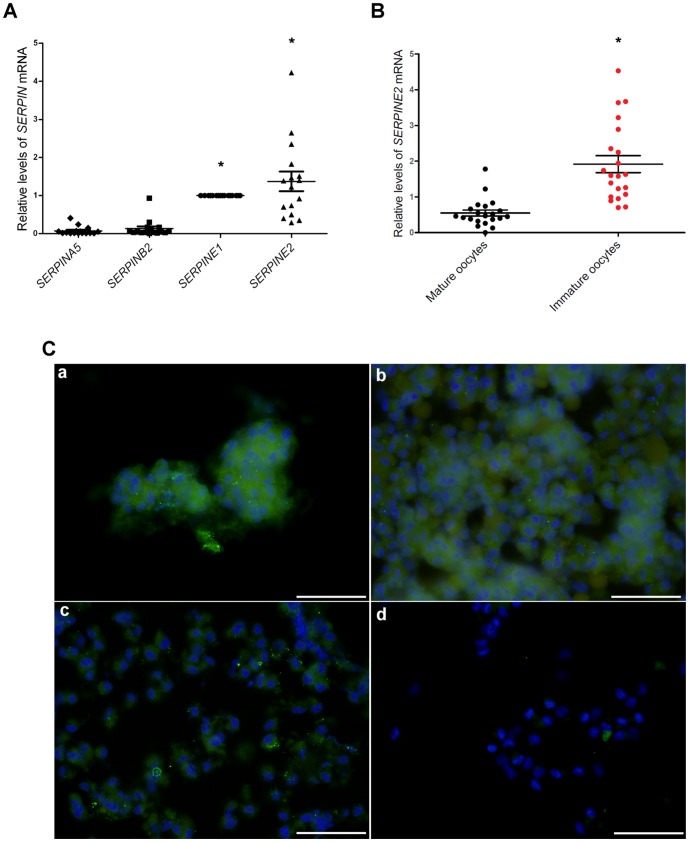
*SERPINE2* expression in cumulus cells of the human oocyte. A, qRT-PCR revealed the relative *SERPIN* mRNA levels in cumulus cells of mature (n = 16) human oocytes. Bars indicate means ± SD of sixteen independent experiments each. *P<0.0001 compared with *SERPINA5* mRNA. B, qRT-PCR indicated *SERPINE2* mRNA levels in cumulus cells of mature (n = 21) and immature (n = 21) human oocytes. Bars indicate means ± SD of twenty-one independent experiments each. *P<0.0001 compared with mature oocytes. C, Representative immunofluorescence staining for SERPINE2 protein levels in the cumulus cells. Cumulus cells collected from immature oocytes at the GV (a) or MI stages (b) and from mature oocytes at the MII stage (c) were immunostained with anti-SERPINE2 antibody, and cumulus cells of MII oocytes were immunostained with the control serum (d). Scale bars, 100 µm.

We analyzed the expression levels of the four *SERPIN*s of the PA system in cumulus cells of the mature human oocyte by qRT-PCR and found that *SERPINE2* were the most highly expressed (Figure 1A). Next, we compared *SERPINE2* mRNA expression levels in cumulus cells collected from mature and immature human oocytes. Cumulus cells from immature oocytes expressed significantly (*P*<0.0001) higher *SERPINE2* mRNA levels than those from mature oocytes (Figure 1B). Similarly, considerably more SERPINE2 protein was detected in cumulus cells from immature human oocytes at the GV and MI stages (Figure 1C, a and b, respectively) than in those from mature MII oocytes or with the control staining of MII oocytes (Figure 1C, c and d, respectively). Other similar cases are shown in Figure S2.

### *Serpine2* and *Plau* were highly expressed in mouse cumulus cells during oocyte maturation

**Figure 2:**
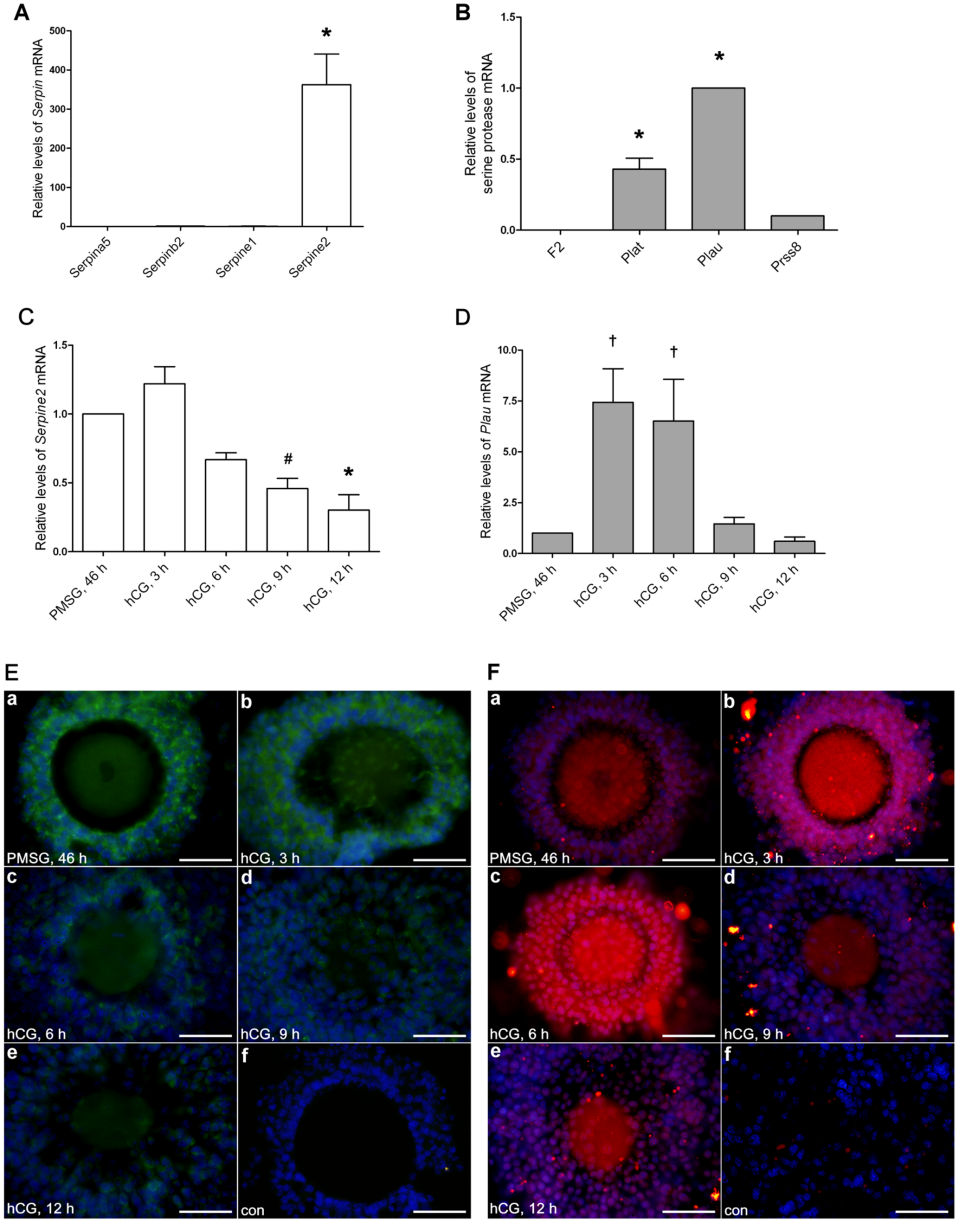
*Serpine2* and *Plau* expression in mouse cumulus cells during oocyte maturation. qRT-PCR revealed the relative mRNA levels of *Serpin*s (A) and serine proteases (B) in cumulus cells surrounding mature oocytes (one mouse for one experiment). Bars indicate means ± SD of three independent experiments each. *P<0.0001 compared with all *Serpin* mRNAs; *P<0.0001 compared with *F2* mRNA. qRT-PCR revealed relative *Serpine2* (C) and *Plau* (D) mRNA levels in mouse cumulus cells surrounding developing oocytes (three mice for each group) during gonadotropin-induced oocyte maturation. Bars indicate means ± SD of three independent experiments each. ^†^ *P*<0.05, ^#^ *P*<0.001, *P<0.0001 compared with the cumulus cells 46 h after PMSG injection alone (PMSG, 46 h). Immunofluorescent staining revealed SERPINE2 (E) and PLAU (F) protein levels in mouse COCs following gonadotropin treatments. COCs were fixed on slides and immunostained using anti-SERPINE2 antiserum and anti-PLAU antibody as described in the Materials and Methods: a, 46 h after PMSG; b, 3 h after hCG; c, 6 h after hCG; d, 9 h after hCG; e, 12 h after hCG administration; f, immunostaining with the control serum (con). Scale bars, 100 µm.

We analyzed the expression profiles of the four *Serpin*s of the PA system in cumulus cells surrounding mature mouse oocytes. Similar to the results with human cumulus cells, *Serpine2* mRNA was the most highly expressed in mouse cumulus cells (Figure 2A). Next, we analyzed the gene expression patterns of SERPINE2-targeted serine proteases in the cumulus cells of mature mouse oocytes using qRT-PCR. *Plau* mRNA was the most highly expressed, followed by *Plat* and *Prss8* mRNAs. *F2* mRNA was almost undetectable (Figure 2B).

To examine the *in vivo* expression pattern of S*erpine2* and *Plau* mRNAs in mouse cumulus cells during oocyte maturation, the cumulus cells were collected at various intervals during gonadotropin-induced oocyte maturation. S*erpine2* mRNA was highly expressed 46 h after PMSG treatment and reached a maximum level 3 h after hCG administration, gradually decreasing to its lowest level 12 h after hCG administration (Figure 2C). *Plau* mRNA was at a low level 46 h after PMSG treatment; however, it peaked 3 h and 6 h after hCG treatment and then gradually decreased to a low level after 12 h (Figure 2D). The relative changes in *Plau* mRNA levels were much greater than the changes in *Serpine2* mRNA levels (Figure 2C and 2D). Expression of SERPINE2 and PLAU proteins was consistent with their mRNA expression in cumulus cells. SERPINE2 was at a relatively high level following PMSG administration and 3 h after hCG treatment (Figure 2E, a and b). PLAU was at a relatively low level after PMSG treatment but peaked approximately 3–6 h after hCG treatment (Figure 2F, a–c). From 6 h after hCG on, SERPINE2 protein levels were gradually decreased to a very lower level (Figure 2E, c–f); on the contrary, PLAU protein levels were still at higher levels at 6 h after hCG (Figure 2F, c) and then sharply decreased to a very low level thereafter (Figure 2F, d–f).

### *Serpine2* silencing or SERPINE2 protein blockage had no effect on cumulus expansion and oocyte maturation *in vitro*

**Figure 3:**
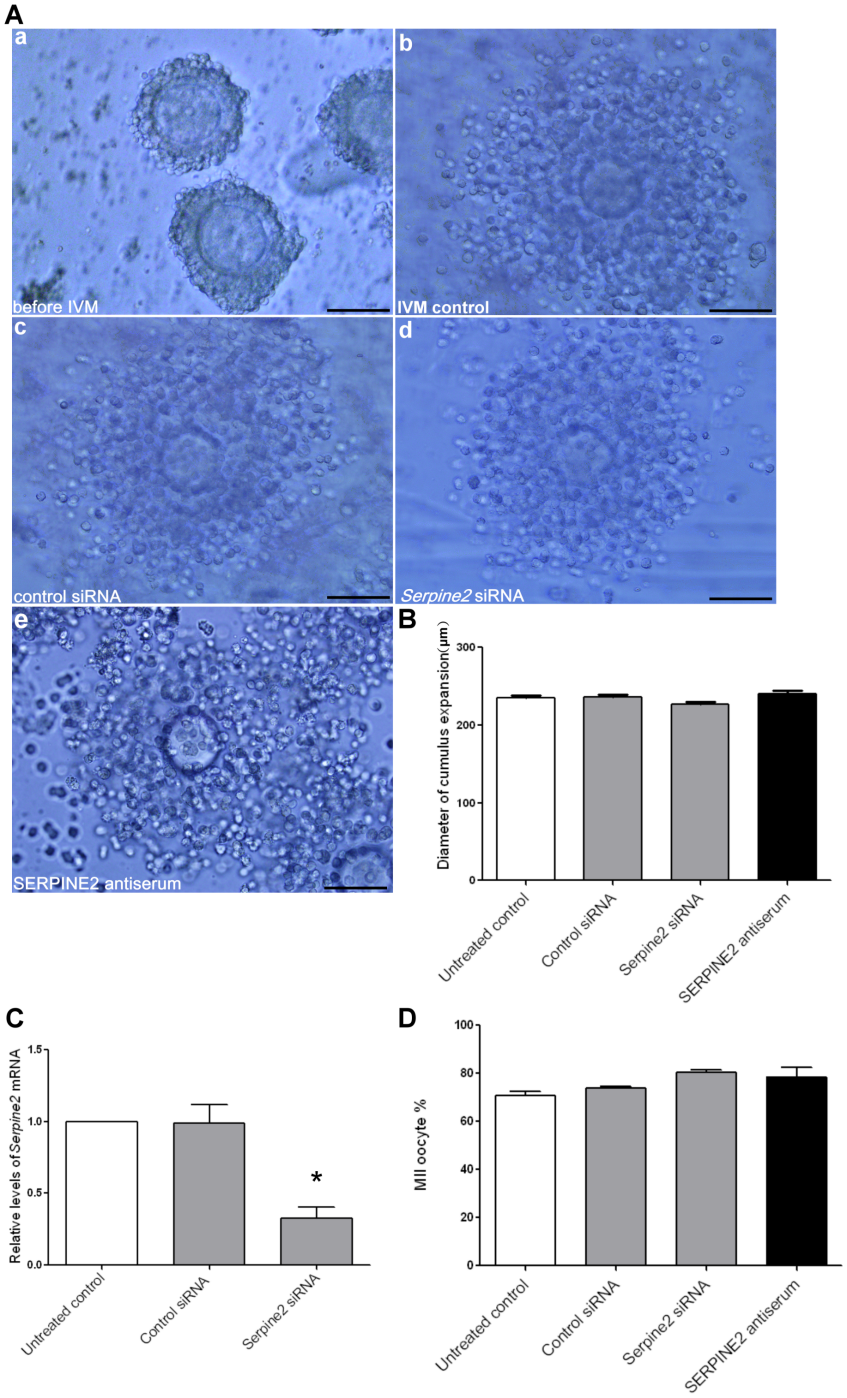
Silencing of *Serpine2* expression and antiserum blockage of SERPINE2 protein. A, The morphologies of COCs isolated from PMSG-primed ovaries (a), cultured in IVM medium for 16 h (b), and treated with control siRNA (c), *Serpine2* siRNA (d), or SERPINE2 antiserum (e) are shown. Scale bars, 100 µm. B, The extent of cumulus expansion in COCs treated as in A (b–e) are presented as the COC diameters after culturing (n = 100 for each). C, qRT-PCR revealed *Serpine2* mRNA levels in mouse cumulus cells with or without *Serpine2* siRNA. *P<0.0001 vs. untreated control. D, MII oocyte maturation rate after 16 h of IVM culture are shown. B–D, Data represent means ± SD of four independent experiments each.

IVM is often used to culture compact immature oocytes collected from PMSG-primed ovaries (Figure 3A, a) for developing MII mature oocytes with fully expanded cumulus cells (Figure 3A, b). To examine the effect of *Serpine2* silencing on oocyte maturation in cumulus cells, siRNA was used to knockdown *Serpine2* mRNA expression during IVM. Cells were also treated with SERPINE2 antiserum to examine the effect of blocking SERPINE2 protein. No detrimental effects on COC structure or morphology (Figure 3A, c–e) or on the extent of cumulus expansion (Figure 3B) were observed, although *Serpine2* mRNA was significantly decreased (*P*<0.0001) and SERPINE2 protein was knocked down by the introduction of *Serpine2* siRNA (Figure 3C and Figure S3, respectively).

The treatments had no effect on oocyte maturation (Table S2). As shown in Figure 3D, more than 70% of oocytes reached the MII stage even when S*erpine2* mRNA in cumulus cells was knocked down. Oocyte maturation was comparable in the media with and without control or *Serpine2* siRNAs or specific anti-SERPINE2 antiserum. Taken together, these findings indicated that eliminating SERPINE2 in cumulus cells had no apparent effect on cumulus expansion and oocyte maturation.

### SERPINE2 overexpression in cumulus cells impaired cumulus expansion and oocyte maturation *in vitro*

**Figure 4:**
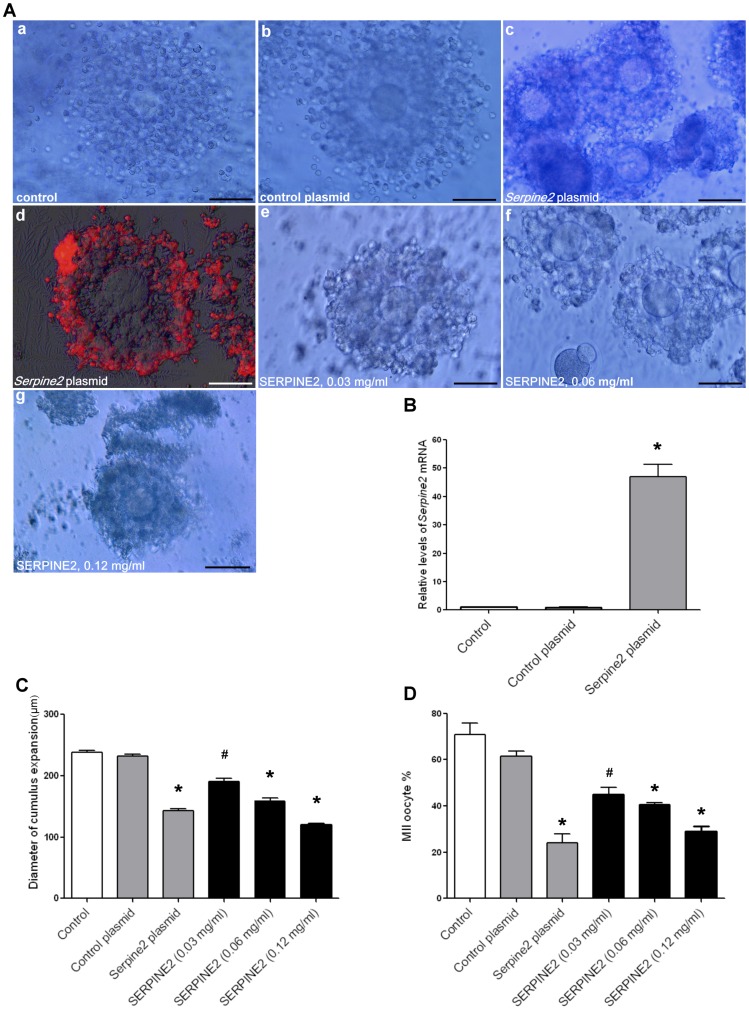
Effects of *Serpine2* overexpression and addition of exogenous SERPINE2 on cumulus expansion. A, The morphologies of untreated and treated COCs after 16 h of IVM culture are shown: a, IVM control; b, transfected with the control plasmid; c, transfected with the *Serpine2*-expressing plasmid. d, Cultured COCs transfected with the *Serpine2*-expressing plasmid were fixed on slides, and SERPINE2 protein expression was monitored by epifluorescence microscopy. The red fluorescence represents coexpression of SERPINE2 and the red fluorescent protein. e–g, COCs were incubated with 0.03, 0.06, and 0.12 mg/ml SERPINE2 protein, respectively. B, qRT-PCR revealed the relative *Serpine2* mRNA levels in cumulus cells transfected with *Serpine2*-expressing or vehicle plasmids. C, The extent of cumulus expansions are shown as COC diameters after 16 h of IVM culture (n = 100 for each group). D, MII oocyte maturation rate after 16 h of IVM culture. B–D, Data represent means ± SD for five independent experiments. ^#^ *P*<0.001, *P<0.0001 vs. untreated control or control plasmid.

To test whether high SERPINE2 levels affected cumulus expansion and oocyte maturation, mouse COCs were transfected with a vector carrying *Serpine2*. The COC morphology was symmetrical with the outward expansion pattern of cumulus cells from the oocyte in both the untreated control and after transfection with control plasmid DNA (Figure 4A, a and b); however, the cumulus cell was compact or in an unexpanded state after transfection with the *Serpine2* plasmid (Figure 4A, c). SERPINE2 protein was significantly overexpressed in cumulus cells after transfection and culturing for 16 h, although most of the protein expression was in the outer layer of cumulus cells (Figure 4A, d). Similarly, exogenously added SERPINE2 resulted in compact, unexpanded cumulus cells that tightly encircled the oocyte (Figure 4A, e–g). *Serpine2* mRNA was significantly overexpressed in cumulus cells after transfection and culturing for 16 h (Figure 4B). *Serpine2* overexpression significantly reduced the extent of cumulus expansion compared to transfection with the control plasmid (*P*<0.0001; Figure 4C, gray bars). Exogenous SERPINE2 also significantly inhibited cumulus cell expansion compared to the control (Figure 4C, black bars).

*Serpine2* overexpression in cumulus cells or exogenously added SERPINE2 significantly reduced oocyte maturation, with most oocytes halting at the MI stage (Table S3). Introduction of the *Serpine2* plasmid into cumulus cells significantly reduced oocyte maturation by approximately 45% compared with the control group (Figure 4D, gray bars). SERPINE2 supplemented exogenously also significantly reduced oocyte maturation by approximately 26–42% (Figure 4D, black bars).

### PLAU protein was involved in cumulus expansion and oocyte maturation

**Figure 5:**
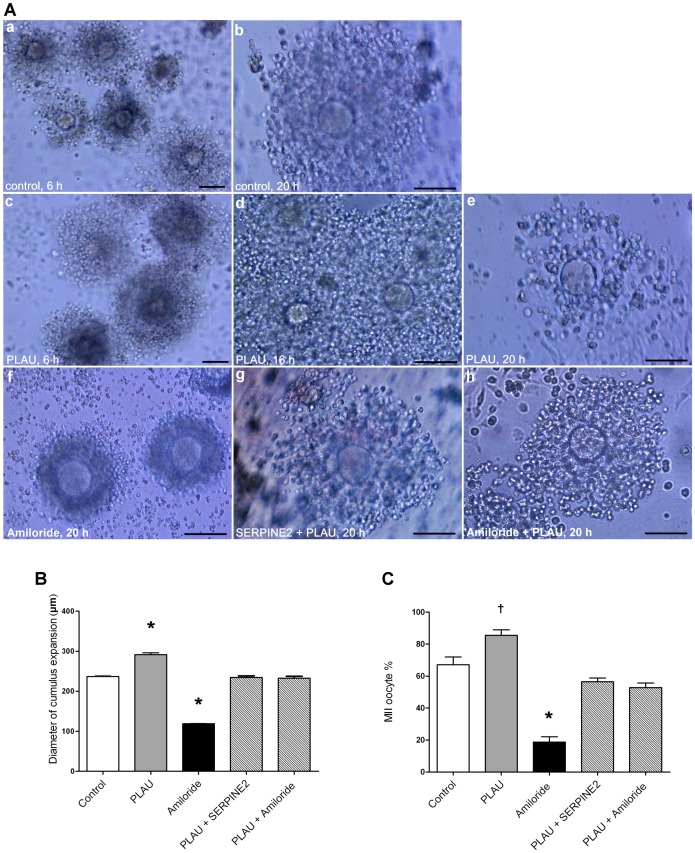
Involvement of PLAU in cumulus expansion and oocyte maturation. A, COC morphologies after IVM culture under varying conditions are shown: a, control COCs after 6 h of culture; b, control COCs after 6 h of culture; c, COCs incubated with 20 U of PLAU for 6 h; d, COCs incubated with 20 U of PLAU for 16 h; e, COCs incubated with 20 U of PLAU for 20 h; f, COCs incubated with 300 µM amiloride for 20 h; g, COCs coincubated with SERPINE2 (0.12 mg/ml) and PLAU (20 U) for 20 h; h, COCs coincubated with amiloride (300 µM) and PLAU (20 U) for 20 h. Scale bars, 100 µm. B, Cumulus expansion was measured as the COC diameters after 16 h of IVM (n = 100 each group). D, MII oocyte maturation rate after 16 h of IVM. B and C, Data represent means ± SD for six independent experiments; ^†^ *P*<0.05, *P<0.0001 vs. controls.

Since PLAU was the most highly expressed serine protease in cumulus cells, we examined PLAU effects on cumulus expansion and oocyte maturation. COC expansion was visible at 6 h of culture (Figure 5A, a) and had fully expanded cumuli with an average diameter of 236 µm after approximately 16–20 h of culture (Figure 5A, b, and 5B, open bar). PLAU supplementation significantly expanded the COCs to an average diameter of 291 µm (Figure 5A, d, and 5B, gray bar), and the expansion occurred earlier at 6 h during IVM compared with that in the control group (Figure 5A, a and c). Furthermore, the PLAU-supplemented cumulus cells degraded earlier (at 20 h) than the control cells (Figure 5A, b and e), which generally degraded at 24 h.

To determine whether the PLAU effect on oocyte maturation was specific, amiloride, a specific inhibitor of PLAU [37], was added to the IVM medium. As shown in Figure 5A, f, and 5B (black bar), cumulus expansion was significantly diminished, and cumulus cells remained encircling the GV oocyte at 20 h, similar to the effects of SERPINE2 addition (Figure 4A, g, and 4C, black bars). To further demonstrate that the inhibition of cumulus expansion was due to PLAU suppression, amiloride or SERPINE2 was coincubated with PLAU during IVM. Intriguingly, the COC morphology appeared normal (Figure 5A, g and h), and the extent of cumulus cell expansion was comparable to that in the control group (Figure 5B, hatched bars).

PLAU significantly promoted oocyte maturation (*P*<0.05), whereas amiloride significantly reduced oocyte maturation (*P*<0.0001) (Figure 5C and Table S4). Coincubation with PLAU and amiloride or SERPINE2 (Figure 5C, hatched bars) reduced maturation to levels comparable with the control group (approximately 53% and 56%, respectively, vs. 67% for the control). Taken together, these data suggested that PLAU was involved in cumulus expansion and oocyte maturation and that its effects could be modulated by SERPINE2.

### Excessive PLAU and SERPINE2 altered matrix gene expression and the hyaluronan status of cumulus cells during IVM

**Figure 6:**
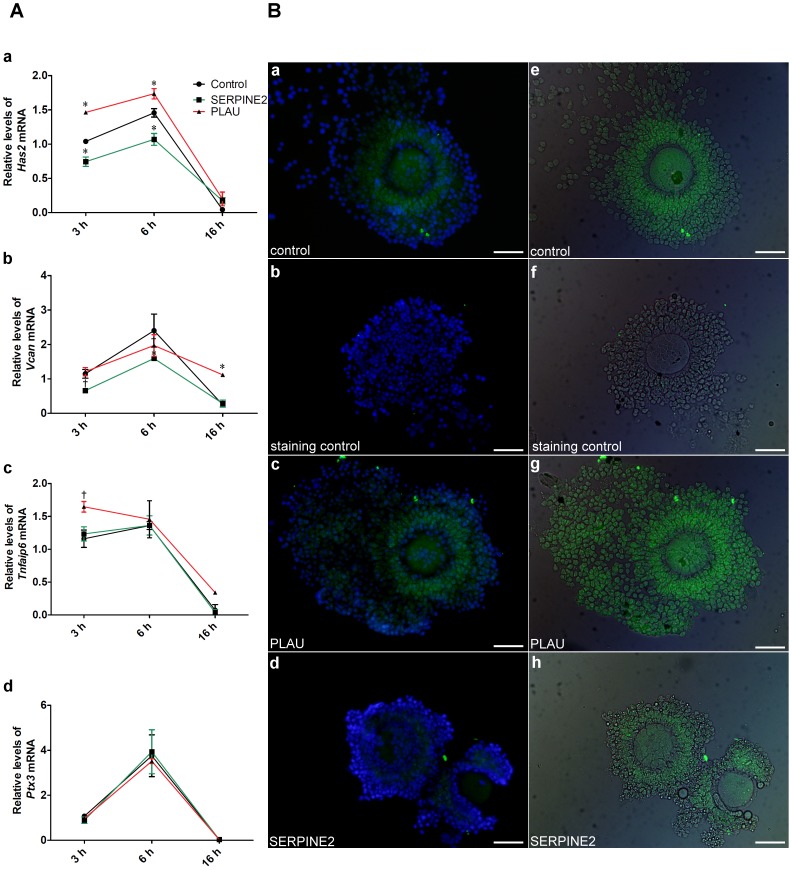
Effect of exogenous PLAU and SERPINE2 on matrix gene expression and the hyaluronan status of cumulus cells during IVM. **COCs were incubated with SERPINE2 (0.06 mg/ml) or PLAU (20 IU) in IVM medium for 3, 6, or 16 h.** After culturing, cumulus cells were collected for qRT-PCR analyses or COCs were transferred onto slides and fixed for hyaluronan evaluation. A, qRT-PCR revealed *Has2* (a), *Vcan* (b), *Tnfaip6*, and *Ptx3* mRNA levels in cumulus cells. Bars indicate means ± SD of three independent experiments each. ^†^ *P*<0.05, *P<0.0001 compared with the group that COCs cultured for 3 h in IVM medium. B, After culturing for 6 h, the hyaluronan contents in untreated COCs (a and e) or COCs treated with PLAU (c and g) or SERPINE2 (d and h) were revealed by treatment with or without (staining control, b and f) HABP (green) as described in Materials and Methods. For contrast, the slides were counterstained with Hoechst 33258 (blue, a–d) or photographed under differential interference contrast microscopy (e–h). Scale bars, 100 µm.

To examine the effect of excessive PLAU and SERPINE2 on the temporal gene expression pattern of the matrix genes in cumulus cells, cumulus cells at 3, 6, and 16 h, the critical time points, during IVM culture were collected from COCs and analyzed by qRT-PCR. PLAU significantly enhanced but SERPINE2 significantly down-regulated cumulus hyaluronan synthase 2 (*Has2*) mRNA levels at 3 and 6 h of IVM compared with that in the control IVM group (Figure 6A, a). *Vcan* mRNA levels in cumulus cells were significantly diminished by exogenous SERPINE2 at 3 and 6 h and by PLAU at 6 h of IVM, but with a PLAU-induced surge at 16 h of IVM (Fig. 6A, b). Cumulus *Tnfaip6* mRNA levels were enhanced by exogenous PLAU after 3 h of IVM; however, this enhancement disappeared at 6 and 16 h of IVM culture. SERPINE2 showed no effect on *Tnfaip6* mRNA expression at all the time points (Fig. 6A, c). Furthermore, exogenous PLAU and SERPINE2 had no effect on *Ptx3* mRNA expression in cumulus cells (Fig. 6A, d).

HAS2 is a critical enzyme required for matrix hyaluronan synthesis [38]. Since *Has2* mRNA expression is the most affected, we analyzed the hyaluronan status of cumulus cells during IVM. After 6 h of culturing, mouse COCs showed moderate expansion with relatively high hyaluronan contents around the cumulus matrix (Fig. 6B, a and e), in contrast to the negative staining control (Fig. 6B, b and f). Hyaluronan staining appeared to cover the entire cell; however, the staining was clearly outside the cumulus cell when ovarian tissue slides were stained. Thus, this staining pattern is probably caused by the steric stacking of cumulus cells (Fig. S4). Intriguingly, exogenous PLAU increased hyaluronan contents (Fig. 6B, c and g), while SERPINE2 supplementation decreased the contents on the cumulus matrix compared with that in the control group (Fig. 6B, d and h).

## Discussion

SERPINE2 overexpression in mouse COCs or its exogenous addition to IVM medium remarkably impaired cumulus expansion, with the COCs exhibiting a compact morphology with a high proportion of GV and MI oocytes, leading to a significant reduction in oocyte maturation (Figure 4 and Table S3). These findings from the mouse model may explain why cumulus cells surrounding immature human oocytes expressed more SERPINE2 than those surrounding mature oocytes and support our hypothesis that aberrantly high SERPINE2 levels correlate with oocyte immaturity.

PLAU and SERPINE2 were the most abundant PA and PA inhibitor, respectively, in murine cumulus cells, and their gene expression levels were coordinately regulated during gonadotropin-induced oocyte maturation (Figure 2). SERPINE2 decreased rapidly 6 h after hCG administration, consistent with apparent cumulus expansion, whereas PLAU remained at a high level. Thus, the net proteolytic activity of PLAU may contribute to the initiation of cumulus expansion. This interplay appears to suggest that the net activity of PLAU may be crucial for cumulus expansion and subsequent oocyte maturation. Furthermore, we found that PLAU depletion via its specific inhibitor, amiloride, largely impaired these biological processes (Figure 4).

Hagglund *et al*. reported high *Serpine2* mRNA levels and low *Plau* mRNA levels in mouse cumulus cells [27] and suggested that SERPINE2 may provide inhibitory activity for protecting the mucified COC matrix from proteolytic degradation. However, we found that cumulus cells expressed both *Plau* and *Serpine2* mRNA and protein during gonadotropin-induced oocyte maturation (Figure 2). Furthermore, *in vivo*, the granulosa cells, which are far more numerous, may also produce these proteins. Hence, we examined their relative expression in cumulus and granulosa cells after hCG 3, 6, and 9 h by immunohistochemistry. Granulosa cells expressed these proteins at levels similar to cumulus cells, especially at 3 and 6 h of hCG treatment (Figure S5). PLAU and SERPINE2 detected in mural granulosa cells may also become associated with the COC matrix during cumulus expansion.

We compared cumulus SERPINE2 and PLAU levels in COCs treated with hCG *in vivo* or cultured *in vitro*. Cumulus SERPINE2 levels had no significant difference at 3 h; however, they were significantly higher at 6 and 9 h after culturing *in vitro* compared with those treated with hCG *in vivo* (Fig. S6). This could be coordinated by FSH and EGF because it was found that FSH could enhance SERPINE2 expression and FSH coupled with EGF (1 ng/ml in this study) could even further enhance SERPINE2 expression in bovine granulosa cells cultured *in vitro* [39]. Furthermore, cumulus PLAU levels were significantly higher at 3 and 6 h when treated with hCG *in vivo* than cultured *in vitro*, despite absence in changes thereafter (Fig. S6). This revealed that PLAU synthesis is repressed in FSH-stimulated COCs by an oocyte-soluble factor [13]. Two cumulus matrix genes, *Adamts1* (a disintegrin and metalloproteinase with thrombospondin-like motifs) and *Vcan*, have higher expression levels in COCs that were treated with hCG *in vivo* than those cultured *in vitro* [40]. These data illustrate that the expression of cumulus matrix genes is altered when COCs are cultured *in vitro*.

Our results may suggest that SERPINE2 has only limited influence or that other inhibitors may be involved in regulating the activity of PLAU and other proteases. This is supported by our result that siRNA silencing of *Serpine2* in cumulus cells had no effect on cumulus expansion or oocyte maturation (Figure 3). Because the siRNA efficiency is still not high enough, we cannot rule out the possibility that the residual SERPINE2 is enough for maintaining normal function. Furthermore, Murer *et al*. reported that *Serpine2* knockout in mice did not result in female infertility [41].

These results appear to indicate that SERPINE2 can be depleted and compensated for by other factors, but that its overexpression hinders cumulus ECM remodeling by proteases and thus impairs cumulus expansion and subsequent oocyte maturation. Cumulus expansion includes limited ECM remodeling and requires sophisticated regulation of proteases and protease inhibitors. A convincingly characterized example is that ADAMTS1 cleavage of VCAM mediates essential remodeling of the COC matrix during ovulation [5].

PA expression in cumulus cells is species-specific. PLAT is the major PA expressed in bovine granulosa cells [42] and rat and human cumulus cells [15], [30] (Figure S7), whereas PLAU is the major PA detected in murine granulosa and cumulus cells [27]. Ny *et al*. found that plasmin activity in the mouse ovary was increased 2–8 h after hCG treatment, and most of the activity was generated by PLAU [43]. D’Alessandris *et al*. found that mouse PLAT and PLAU activities dramatically increased between 16 h and 20 h of IVM and concluded that both PAs might function to destabilize the expanded COC matrix [13]. Several studies also demonstrated that PAs play a crucial role in follicle wall rupture during ovulation [13]-[17]; however, their function in oocyte maturation during pre-ovulation remained unclear. Our study provides the first evidence that PLAU and its inhibitor SERPINE2 are involved in murine cumulus expansion and oocyte maturation.

Cumulus matrix genes, e.g., *Has2* and *Vcan*, are induced and these genes normally peak around 4–6 h after treatment with hCG *in vivo* or FSH/EGF *in vitro* [40]. The temporal cumulus matrix *Has2* and *Vcan* expression and hyaluronan status were altered by exogenous supplementation of SERPINE2 and PLAU during IVM (Figure 6). *Has2* expression is correlated with hyaluronan synthesis, which is necessary for cumulus expansion [44]. SERPINE2 down-regulated *Has2* expression; thus, reducing the matrix hyaluronan contents. VCAM, an important cumulus matrix proteoglycan, is involved in cumulus expansion [5]. SERPINE2 also down-regulated *Vcan* expression at critical time points of cumulus expansion during IVM. These findings may explain why excessive SERPINE2 resulted in compact, unexpanded cumulus cells (Figure 4A). On the other hand, PLAU up-regulated *Has2* expression and increased hyaluronan contents. These effects may cause earlier expansion and degradation than that seen in the control group (Figure 5A).

Many hyaluronan-binding proteins, e.g., ITIH [1], PTX3 [2], [3], TNFAIP6 [4], and VCAM [5], as well as their interactions [2], [6], [7], have been shown to be crucial for cumulus structural integrity. We here demonstrate that two new components, SERPINE2 and PLAU, expressed in the cumulus ECM play roles in cumulus expansion and oocyte maturation.

PLAU supplemented exogenously led to early cumulus expansion and matrix degradation and enhanced oocyte maturation (Figure 5). This is probably through up-regulation of *Has2* expression and increased hyaluronan contents in the cumulus matrix (Figure 6). Coincubation of PLAU with amiloride or SERPINE2 neutralized the PLAU effects on cumulus expansion and subsequent oocyte maturation. These findings indicate that SERPINE2 can modulate PLAU activity. Superovulation treatments sometimes retrieve immature oocytes from assisted reproductive technology (ART) patients [45], [46]. Thus, PLAU supplemented exogenously may be helpful for maturation of immature oocytes during IVM.

In summary, the present results support the involvement of SERPINE2 and its cognate serine protease PLAU in cumulus expansion and subsequent oocyte maturation. Depletion or elimination of SERPINE2 expression has no effect on cumulus expansion and oocyte maturation; however, high SERPINE2 levels bound to the cumulus ECM could down-regulate *Has2* and *Vcan* expression and decrease matrix hyaluronan contents, leading to suppressed cumulus expansion and oocyte maturation. On the other hand, PLAU supplementation to IVM culture medium up-regulated *Has2* expression and increased matrix hyaluronan contents could be a potential therapeutic strategy for rescuing immature human oocytes collected during ART procedures, although further study is required.

## Supporting Information

Figure S1 **Construction of the** ***Serpine2*** **expression vector.** (a) A DNA fragment containing MCS and DsRed2 of the pIRES2-DsRed2 vector was amplified using primer pairs containing *EcoR*I sites. (b) The pCX-EGFP vector was digested with *EcoR*I. (c) The DNA fragment from (a) was ligated into the *EcoR*I-digested pCX-EGFP vector to form an intermediate vector pCX-DsRed2. (d) A PCR-amplified full-length *Serpine2* cDNA propagated by TA cloning was digested with *Xma*I and cloned into the pCX-DsRed2 vector to create pCX-Serpine2-DsRed2.
(TIF)

Figure S2 **Immunofluorescence staining for SERPINE2 protein levels in human cumulus cells.** COCs were collected from patients whose oocytes had all 3 nuclear stages (GV, MI, MII). Cumulus cells collected from immature oocytes at the GV (a) or MI stages (b) and from mature oocytes at the MII stages (c) were immunostained with anti-SERPINE2 antibody (green for case 2 and 3 or red for case 4), and cumulus cells of MII oocytes were immunostained with the control serum (d). For contrast, the slides were counterstained with Hoechst 33258 (blue, case 2 and 3) or hematoxylin (blue, case 4). Scale bars, 100 µm.
(TIF)

Figure S3 **Immunohistochemistry of SERPINE2 protein in cumulus cells treated with** ***Serpine2*** **siRNA.** COCs treated without (control, a and e) or with control siRNA (b and f), or *Serpine2* siRNA (c and g) were cultured in IVM medium for 16 h. After culturing, COCs were transferred and fixed onto slides and immunostained by anti-SERPINE2 (green) or control antiserum (d and h). For contrast, the slides were counterstained with Hoechst 33258 (blue, a–d) or photographed under differential interference contrast microscopy (e–h). Scale bars, 100 µm.
(TIF)

Figure S4 **Hyaluronan matrix staining of the tissue section and COC.** The hyaluronan on ovarian sections that were PMSG-primed and treated with hCG for 3 h (a) or COCs that were cultured for 6 h *in vitro* and then transferred onto slides (b) were stained with HABP (green) as described in Materials and Methods. For contrast, the slides were counterstained with Hoechst 33258 (H33258, blue, c and d) and the merged images are also shown (e and f). Scale bars, 100 µm.
(TIF)

Figure S5 **Immunolocalization of SERPINE2 and PLAU in ovarian follicles during gonadotropin treatment.** Ovarian sections from PMSG-primed and hCG administration for 3, 6, and 9 h were immunostained using anti-SERPINE2 antiserum and anti-PLAU antibody as described in Materials and Methods: a and e, 3 h after hCG; b and f, 6 h after hCG; c and g, 9 h after hCG; d and h, immunostaining with the control serum (con). Scale bars, 100 µm.
(TIF)

Figure S6 **Cumulus SERPINE2 and PLAU protein levels in COCs treated with hCG** ***in vivo*** **or cultured** ***in vitro*** **.** COCs isolated from PMSG-primed ovaries (a and f), treated with hCG for 3, 6, 9, and 12 h (b–e, respectively) or IVM culture for 3, 6, 9, and 16 h (g–j, respectively), were immunostained using anti-SERPINE2 antiserum and anti-PLAU antibody as described in Materials and Methods. The slides counterstained with Hoechst 33258 were photographed using a fluorescence microscope (Olympus BX 40) equipped with an Olympus DP-70 digital camera. The percentage of positively stained cells was determined using TissueQuest software (TissueGnostics, Vienna, Austria). A chi-square test was performed to independently compare the significance of difference in expression levels of SERPINE2 or PLAU in cumulus cells at different time points. *P<0.0001 compared with *in vivo* and *in vitro* samples at the same time point. Scale bars, 100 µm.
(TIF)

Figure S7 ***PLAT*** **expression in cumulus cells of human oocytes.** qRT-PCR revealed the relative levels of serine protease mRNAs in cumulus cells of mature (n = 16) human oocytes. Bars indicate means ± SD of sixteen independent experiments each. *P<0.0001 compared with *F2* mRNA.
(TIF)

Table S1 Summary of real-time PCR primers.
(DOC)

Table S2 Effects of *Serpine2* siRNA and anti-SERPINE2 antiserum on oocyte maturation.
(DOC)

Table S3 Effects of *Serpine2* overexpression in cumulus cells and exogenously added SERPINE2 on oocyte maturation.
(DOC)

Table S4 Effects of PLAU protein on oocyte maturation.
(DOC)
