# Supplementary figures and images for: Involvement of the Serine Protease Inhibitor, SERPINE2, and the Urokinase Plasminogen Activator in Cumulus Expansion and Oocyte Maturation
Source: PLoS One. 2013 Aug 30;8(8):e74602. doi: 10.1371/journal.pone.0074602 (PMC3758271; doi:10.1371/journal.pone.0074602)

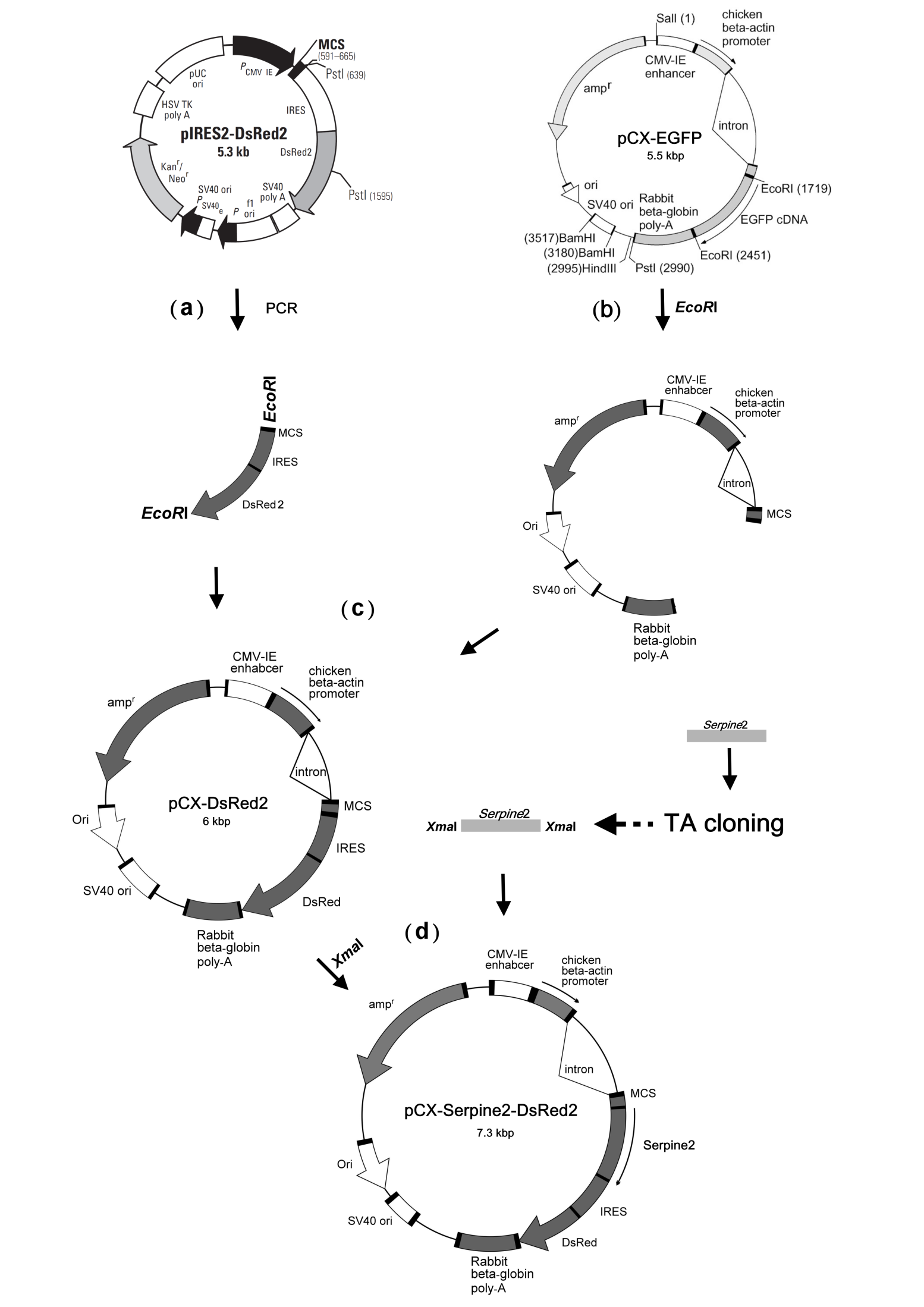

Supplement: Figure S1 — Construction of the Serpine2 expression vector. (a) A DNA fragment containing MCS and DsRed2 of the pIRES2-DsRed2 vector was amplified using primer pairs containing EcoRI sites. (b) The pCX-EGFP vector was digested with EcoRI. (c) The DNA fragment from (a) was ligated into the EcoRI-digested pCX-EGFP vector to form an intermediate vector pCX-DsRed2. (d) A PCR-amplified full-length Serpine2 cDNA propagated by TA cloning was digested with XmaI and cloned into the pCX-DsRed2 vector to create pCX-Serpine2-DsRed2. (TIF) [file pone.0074602.s001.tif]

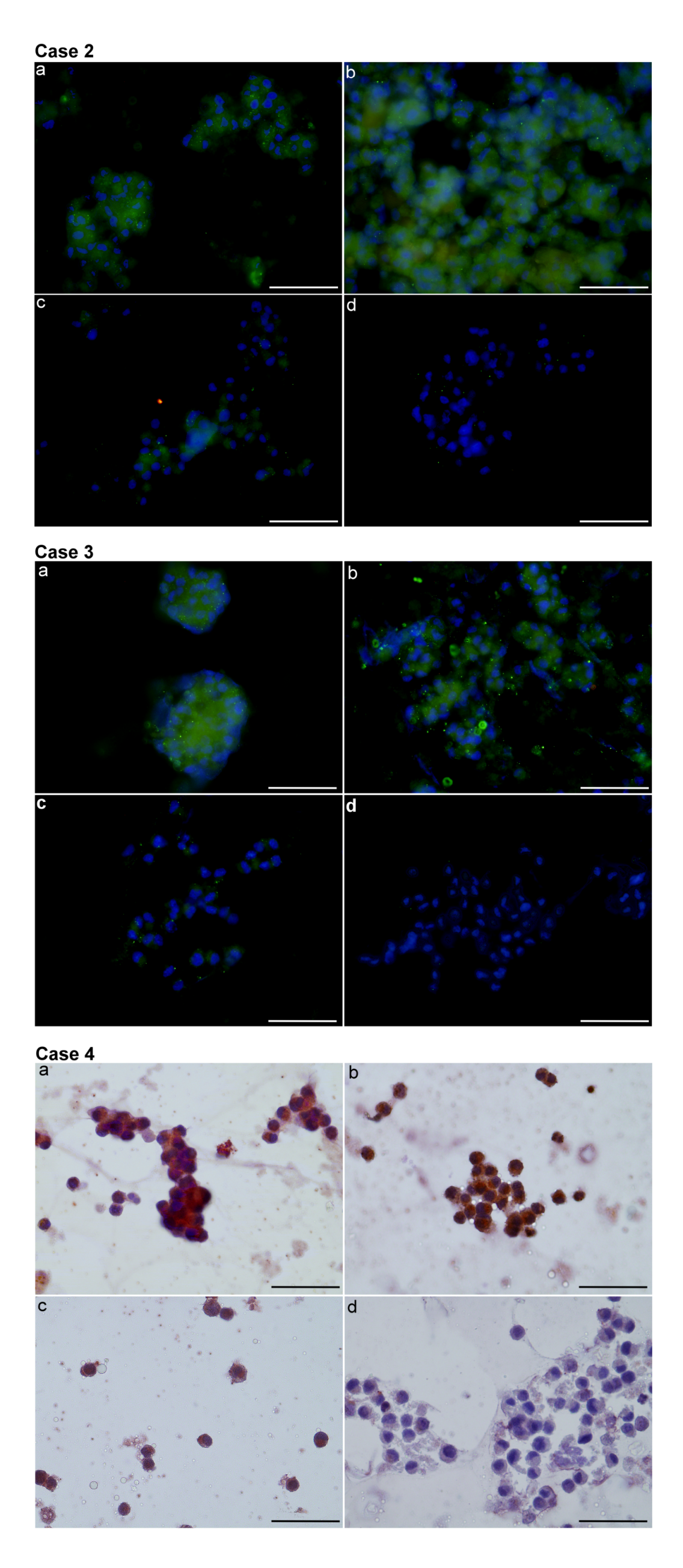

Supplement: Figure S2 — Immunofluorescence staining for SERPINE2 protein levels in human cumulus cells. COCs were collected from patients whose oocytes had all 3 nuclear stages (GV, MI, MII). Cumulus cells collected from immature oocytes at the GV (a) or MI stages (b) and from mature oocytes at the MII stages (c) were immunostained with anti-SERPINE2 antibody (green for case 2 and 3 or red for case 4), and cumulus cells of MII oocytes were immunostained with the control serum (d). For contrast, the slides were counterstained with Hoechst 33258 (blue, case 2 and 3) or hematoxylin (blue, case 4). Scale bars, 100 µm. (TIF) [file pone.0074602.s002.tif]

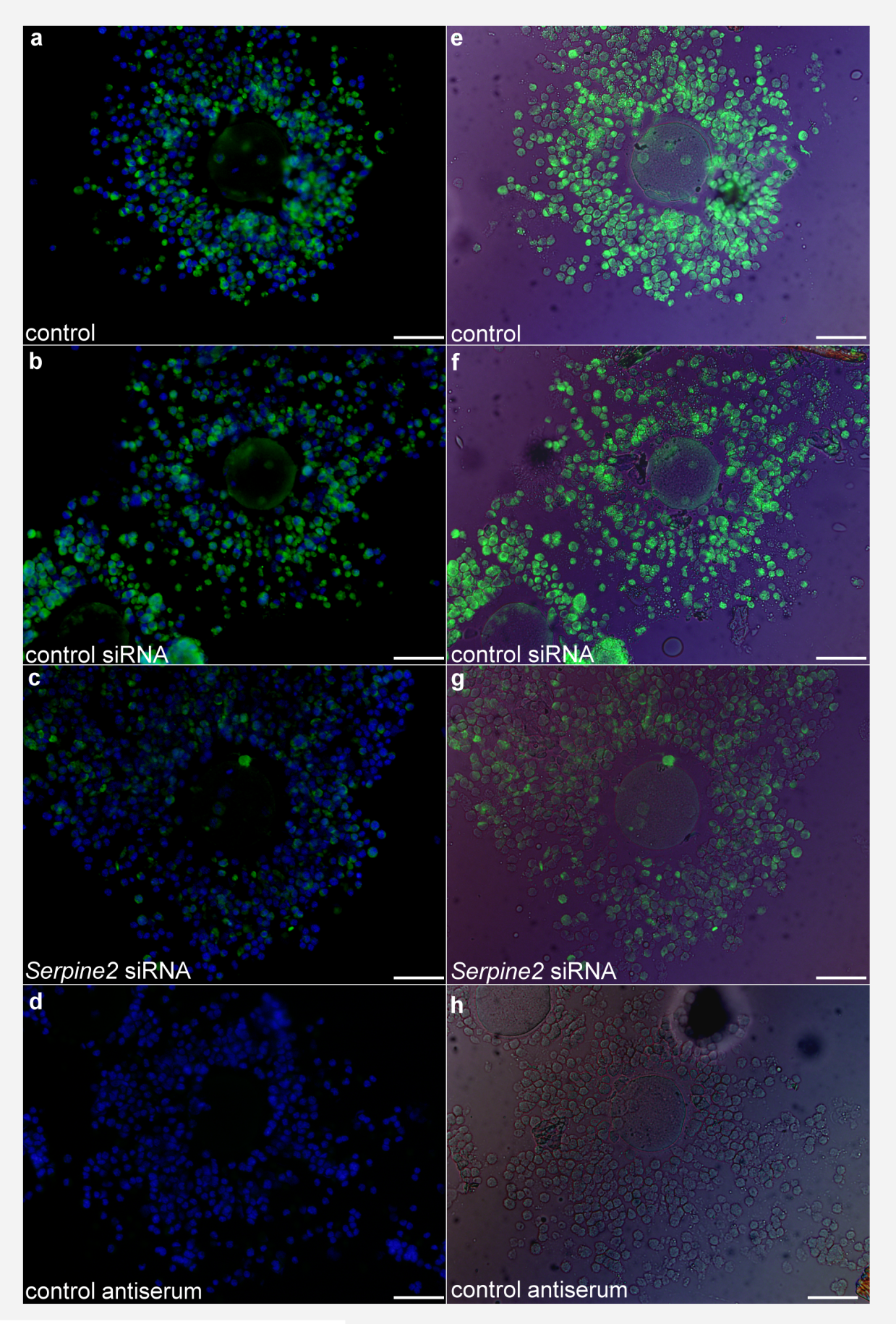

Supplement: Figure S3 — Immunohistochemistry of SERPINE2 protein in cumulus cells treated with Serpine2 siRNA. COCs treated without (control, a and e) or with control siRNA (b and f), or Serpine2 siRNA (c and g) were cultured in IVM medium for 16 h. After culturing, COCs were transferred and fixed onto slides and immunostained by anti-SERPINE2 (green) or control antiserum (d and h). For contrast, the slides were counterstained with Hoechst 33258 (blue, a–d) or photographed under differential interference contrast microscopy (e–h). Scale bars, 100 µm. (TIF) [file pone.0074602.s003.tif]

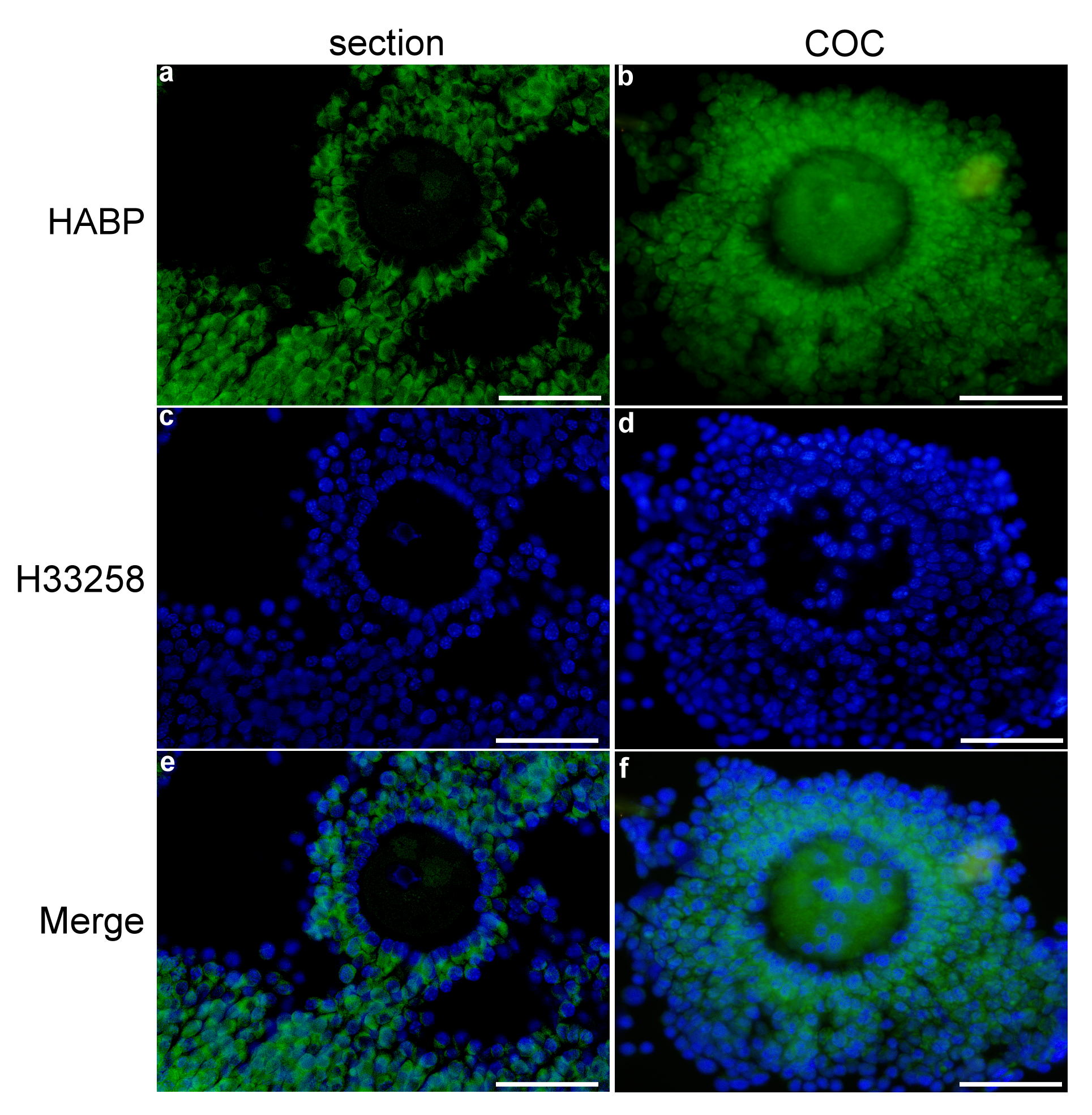

Supplement: Figure S4 — Hyaluronan matrix staining of the tissue section and COC. The hyaluronan on ovarian sections that were PMSG-primed and treated with hCG for 3 h (a) or COCs that were cultured for 6 h in vitro and then transferred onto slides (b) were stained with HABP (green) as described in Materials and Methods. For contrast, the slides were counterstained with Hoechst 33258 (H33258, blue, c and d) and the merged images are also shown (e and f). Scale bars, 100 µm. (TIF) [file pone.0074602.s004.tif]

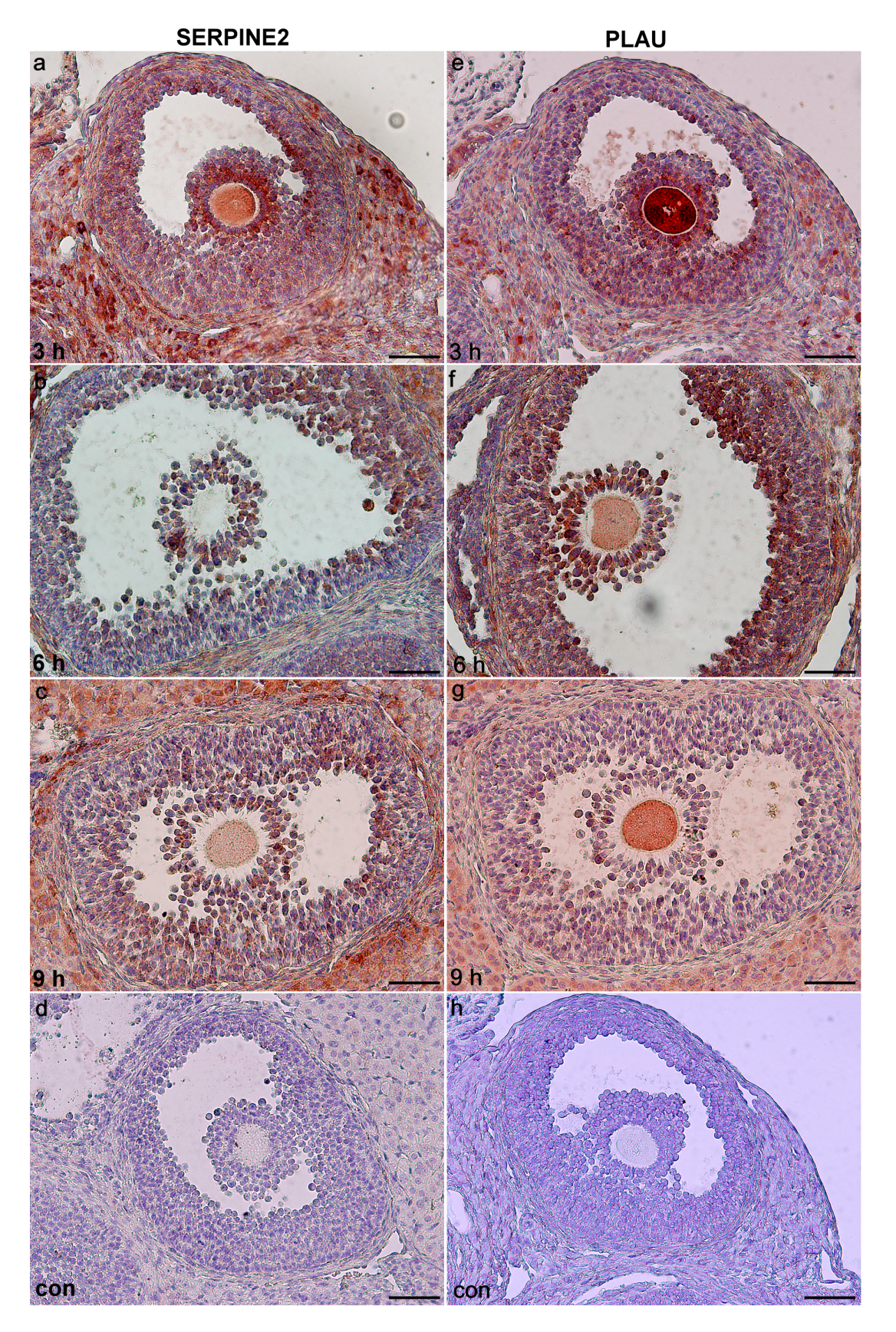

Supplement: Figure S5 — Immunolocalization of SERPINE2 and PLAU in ovarian follicles during gonadotropin treatment. Ovarian sections from PMSG-primed and hCG administration for 3, 6, and 9 h were immunostained using anti-SERPINE2 antiserum and anti-PLAU antibody as described in Materials and Methods: a and e, 3 h after hCG; b and f, 6 h after hCG; c and g, 9 h after hCG; d and h, immunostaining with the control serum (con). Scale bars, 100 µm. (TIF) [file pone.0074602.s005.tif]

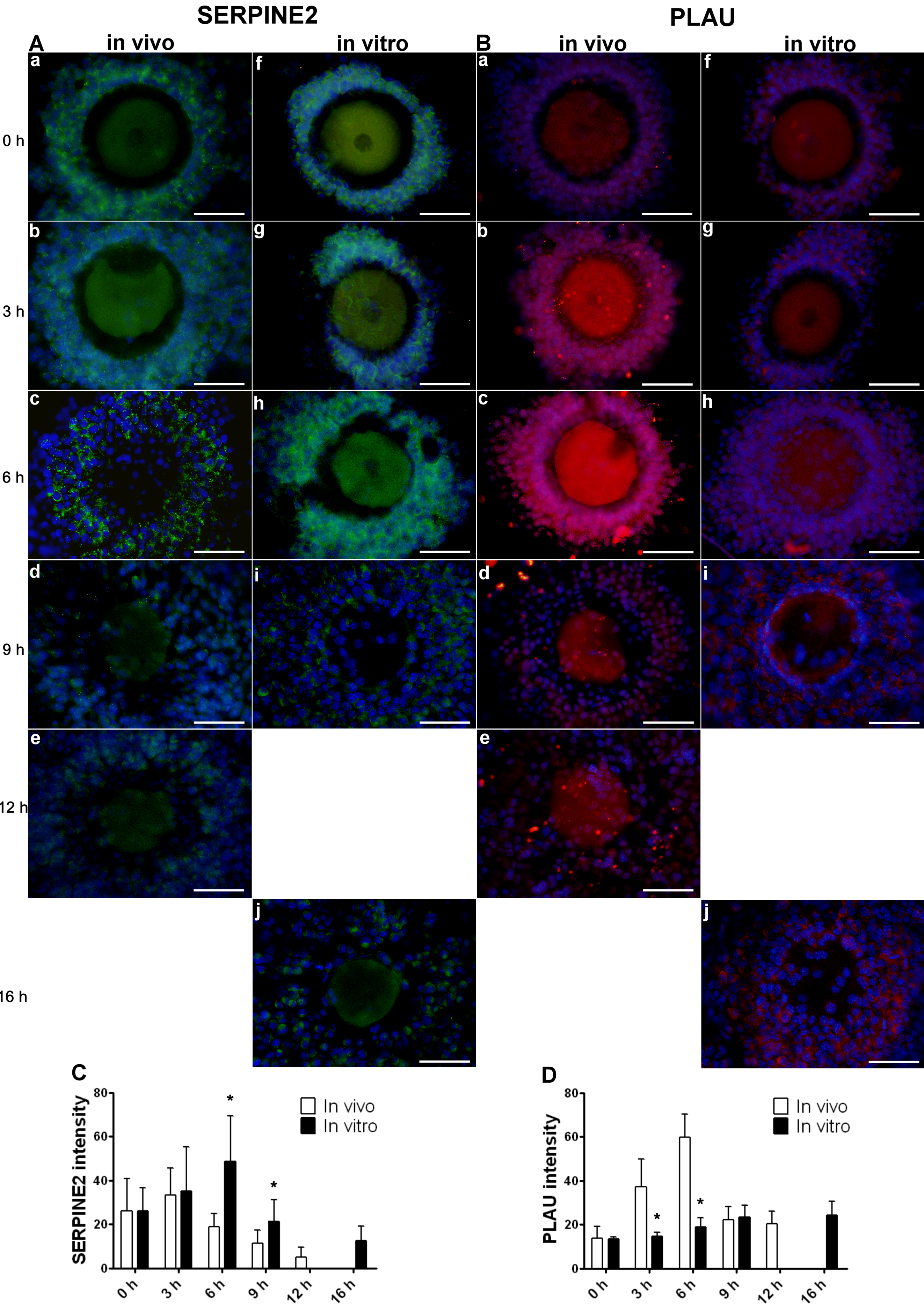

Supplement: Figure S6 — Cumulus SERPINE2 and PLAU protein levels in COCs treated with hCG in vivo or cultured in vitro . COCs isolated from PMSG-primed ovaries (a and f), treated with hCG for 3, 6, 9, and 12 h (b–e, respectively) or IVM culture for 3, 6, 9, and 16 h (g–j, respectively), were immunostained using anti-SERPINE2 antiserum and anti-PLAU antibody as described in Materials and Methods. The slides counterstained with Hoechst 33258 were photographed using a fluorescence microscope (Olympus BX 40) equipped with an Olympus DP-70 digital camera. The percentage of positively stained cells was determined using TissueQuest software (TissueGnostics, Vienna, Austria). A chi-square test was performed to independently compare the significance of difference in expression levels of SERPINE2 or PLAU in cumulus cells at different time points. *P<0.0001 compared with in vivo and in vitro samples at the same time point. Scale bars, 100 µm. (TIF) [file pone.0074602.s006.tif]

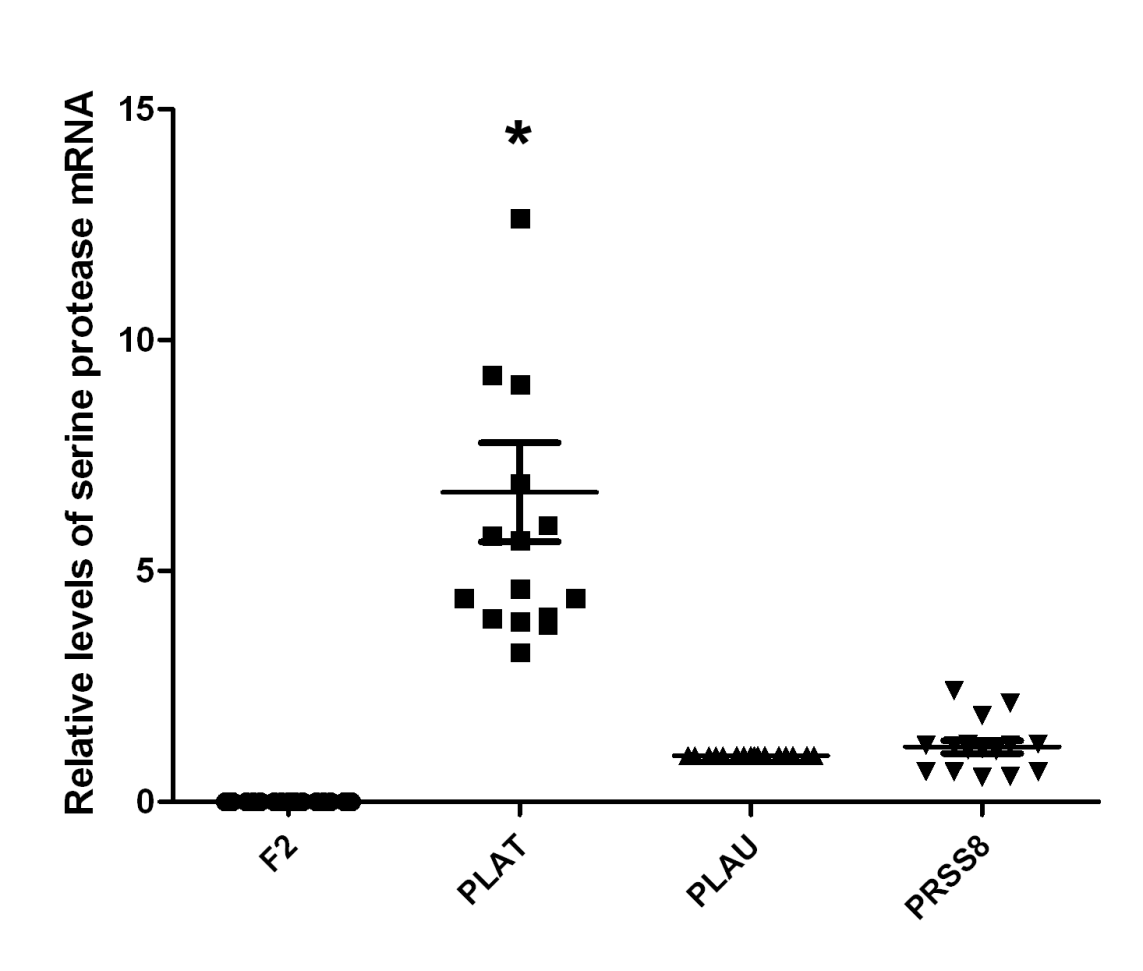

Supplement: Figure S7 — PLAT expression in cumulus cells of human oocytes. qRT-PCR revealed the relative levels of serine protease mRNAs in cumulus cells of mature (n = 16) human oocytes. Bars indicate means ± SD of sixteen independent experiments each. *P<0.0001 compared with F2 mRNA. (TIF) [file pone.0074602.s007.tif]
