# Supplementary material for: Involvement of the Serine Protease Inhibitor, SERPINE2, and the Urokinase Plasminogen Activator in Cumulus Expansion and Oocyte Maturation
Source: PLoS One. 2013 Aug 30;8(8):e74602. doi: 10.1371/journal.pone.0074602 (PMC3758271; doi:10.1371/journal.pone.0074602)
Supplement: Table S1 — Summary of real-time PCR primers. (DOC) [file pone.0074602.s008.doc]

**Table S1.** Summary of real-time PCR primers

| **Gene^a^** | **Primer** | **Sequence** | **Position** | **Product size (bp)** |
| --- | --- | --- | --- | --- |
| Human | | | | |
| *SERPINA5* | F^b^ | 5’-CCCACACGACTGCAACATACAG-3’ | 1585-1606 | 118 |
|  | R^c^ | 5’-TGAGCAAGTCATCCAGCCTCTC-3' | 1702-1681 |  |
| *SERPINB2* | F | 5’-TTCTGAAGTGTTCCACCAAGCC-3’ | 1386-1407 | 100 |
|  | R | 5’-TCCATGTCCAGTTCTCCCTGTC-3’ | 1485-1464 |  |
| *SERPINE1* | F | 5’-TCTGTTCCAGTCACATTGCCAT-3’ | 1767-1788 | 115 |
|  | R | 5’-TGCCACAGTGGACTCTGAGATG-3’ | 1881-1860 |  |
| *SERPINE2* | F | 5’-TCTCATTGCAAGATCATCGCC-3’ | 1323-1343 | 97 |
|  | R | 5’-CCCCATGAATAACACAGCACC-3’ | 1419-1399 |  |
| *F2* | F | 5’-ATCCGCATCACTGACAACATG-3’ | 1667-1687 | 135 |
|  | R | 5’-CCCATTTGATACCAGCGGTT-3’ | 1801-1782 |  |
| *PLAT* | F | 5’-TCAGCTAAAGCCCAACCTCCT-3’ | 2055-2075 | 124 |
|  | R | 5’-CTAATGCAATCCGTCTTTCCTG-3’ | 2178-2157 |  |
| *PLAU* | F | 5’-TCACCACAACGACATTGCCTT-3’ | 959-979 | 134 |
|  | R | 5’-TGATCTCACAGCTTGTGCCAAA-3’ | 1092-1071 |  |
| *PRSS8* | F | 5’-TTCCCTGATGGCCTTTGGA-3’ | 1419-1437 | 120 |
|  | R | 5’-CCCAAAAAGCACACCCAGAAG-3’ | 1538-1518 |  |
| *RPL19* | F | 5’-TCAGCTTGTGGATGTGTTCCA-3’ | 406-426 | 93 |
|  | R | 5’-TCGATCGCCACATGTATCACAG-3’ | 498-477 |  |
| Mouse | | | | |
| *Serpina5* | F | 5’-TCTCCATTGAGGCTACCTACAAACT-3’ | 1071-1095 | 131 |
|  | R | 5’-GTGCACCATCTCAGACAACTTGA-3’ | 1201-1179 |  |
| *Serpinb2* | F | 5’-TTCCGTGTGAACTCGCATGA-3’ | 664-683 | 143 |
|  | R | 5’-GGAAGCAACAGGAGCATGCT-3’ | 806-787 |  |
| *Serpine1* | F | 5’-CAGAGCAACAAGTTCAACTACACTGA-3’ | 810-835 | 106 |
|  | R | 5’-CAGCGATGAACATGCTGAGG-3’ | 915-896 |  |
| *Serpine2* | F | 5’-CAGATCATCAAGTCACGGCCT-3’ | 269-289 | 119 |
|  | R | 5’-ACCGTGGAGAGCTGCTTCTTT-3’ | 387-367 |  |
| *F2* | F | 5’-TTCTGTGCTGGCTTCAAGGTG-3’ | 1654-1674 | 115 |
|  | R | 5’-ACCCATTTGATACCAGCGGTT-3’ | 1768-1748 |  |
| *Plat* | F | 5’-AAGAGAGCAGCTCTGTTGGCAC-3’ | 1366-1387 | 112 |
|  | R | 5’-AATGGAGACGATGCCTCATGC-3’ | 1477-1457 |  |
| *Plau* | F | 5’-GAAGCGACCCTGGTGCTATG-3’ | 445-464 | 82 |
|  | R | 5’-TTTGCTAAGAGAGCAGTCATGCA-3’ | 526-504 |  |
| *Prss8* | F | 5’-AAGCTGTGACCATTCTGCTCCT-3’ | 117-138 | 63 |
|  | R | 5’-CAGTCCCGTCAGCTCGGA-3’ | 179-162 |  |
| *Has2* | F | 5’-AAGACCCTATGGTTGGAGGTG-3’ | 1245-1265 | 167 |
|  | R | 5’-CATTCCCAGAGGACCGCTTAT-3’ | 1411-1391 |  |
| *Vcan* | F | 5’-AACCAGGCGCTGATCCTTAAA-3’ | 10499-10519 | 129 |
|  | R | 5’-CGGCAGTCCCATAATCCAAAC-3’ | 10627-10607 |  |
| *Tnfaip6* | F | 5’-GATGGTCGTCCTCCTTTGCTT-3’ | 63-83 | 141 |
|  | R | 5’-TATCTGCCAGCCCGAGCTT-3’ | 203-185 |  |
| *Ptx3* | F | 5’-GGACAACGAAATAGACAATGGACTT-3’ | 265-289 | 109 |
|  | R | 5’-CGAGTTCTCCAGCATGATGAAC-3’ | 373-352 |  |
| *Hprt* | F | 5’-GAATCACGTTTGTGTCATTAGTGAAA-3’ | 752-777 | 62 |
|  | R | 5’-TGCGCTCATCTTAGGCTTTGTA-3’ | 813-792 |  |

^a^ GenBank accession nos.: *SERPINA5*, NM_000624; *SERPINB2*, NM_001143818; *SERPINE1*, NM_000602; *SERPINE2*, NM_001136528; *F2*, NM_000506; *PLAT*, NM_000930; *PLAU*, NM_001145031; *PRSS8*, NM_002773; *RPL19*, NM_000981; *Serpina5*, NM_172953; *Serpinb2*, NM_011111; *Serpine1*, NM_008871; *Serpine2*, NM_009255; *F2*, NM_010168; *Plat*, NM_008872; *Plau*, NM_008873; *Prss35*, NM_178738; *Has2*, NM_008216.3; Vcan, NM_001081249.1; *Tnfaip6*, NM_009398.2; Ptx3, NM_008987.3; *Hprt*, NM_013556.

^b^ F, forward primer.

^c^ R, reverse primer.
