# Supplementary material for: Involvement of the Serine Protease Inhibitor, SERPINE2, and the Urokinase Plasminogen Activator in Cumulus Expansion and Oocyte Maturation
Source: PLoS One. 2013 Aug 30;8(8):e74602. doi: 10.1371/journal.pone.0074602 (PMC3758271; doi:10.1371/journal.pone.0074602)
Supplement: Table S2 — Effects of Serpine2 siRNA and anti-SERPINE2 antiserum on oocyte maturation. (DOC) [file pone.0074602.s009.doc]

**Table S2.** Effects of *Serpine2* siRNA and anti-SERPINE2 antiserum on oocyte maturation

| Oocyte stage | Control (%) | Control siRNA (%) | *Serpine2* siRNA (%) | SERPINE2 antiserum (%) |
| --- | --- | --- | --- | --- |
| GV | 8.49 ± 3.51 | 4.26 ± 1.74 | 4.64 ± 3.28 | 3.71 ± 1.58 |
| MI | 20.67 ± 2.33 | 22.04 ± 2.91 | 15.10 ± 3.26 | 19.96 ± 2.90 |
| MII | 70.84 ± 2.78 | 73.69 ± 2.51 | 78.42 ± 3.9 | 78.42 ± 1.97 |
| Number of COCs | 125 | 138 | 155 | 379 |

GV, germinal vesicle; MI, metaphase I; MII, metaphase II; COCs, cumulus–oocyte complexes.

Data are means ± SD of three (groups of control, control siRNA, and Serpine2 siRNA) and four (the group of SERPINE2 antiserum) independent experiments. Percentages are based on the total number of oocytes examined.
