# Supplementary material for: Involvement of the Serine Protease Inhibitor, SERPINE2, and the Urokinase Plasminogen Activator in Cumulus Expansion and Oocyte Maturation
Source: PLoS One. 2013 Aug 30;8(8):e74602. doi: 10.1371/journal.pone.0074602 (PMC3758271; doi:10.1371/journal.pone.0074602)
Supplement: Table S3 — Effects of Serpine2 overexpression in cumulus cells and exogenously added SERPINE2 on oocyte maturation. (DOC) [file pone.0074602.s010.doc]

**Table S3.** Effects of *Serpine2* overexpression in cumulus cells and exogenously added SERPINE2 on oocyte maturation

| Oocyte  stage | Control (%) | Control plasmid (%) | *Serpine2* plasmid (%) | SERPINE2**^a^** (%) | SERPINE2**^b^**  (%) | SERPINE2**^c^**  (%) |
| --- | --- | --- | --- | --- | --- | --- |
| GV | 7.14 ± 2.14 | 8.39 ± 0.65 | 9.52 ± 1.42 | 10.31 ± 2.28 | 7.35 ± 1.42 | 8.95 ± 2.34 |
| MI | 22.10 ± 1.50 | 17.35 ± 6.06 | 39.76 ± 8.59 | 44.67 ± 7.37 | 52.19 ± 3.22 | 62.03 ± 1.71 |
| MII | 70.76 ± 8.54 | 61.40 ± 5.40 | 26.07 ± 8.95^*^ | 45.02 ± 5.31^#^ | 40.46 ± 1.84^*^ | 29.01 ± 3.94^*^ |
| Number of COCs | 108 | 154 | 142 | 633 | 783 | 367 |

GV, germinal vesicle; MI, metaphase I; MII, metaphase II; COCs, cumulus–oocyte complexes.

^a–c^ SERPINE2 at 0.03, 0.06, and 0.12 mg/ml, respectively.

Data are means ± SD of three (groups of control, control plasmid, and *Serpine2* plasmid) and five (groups of SERPINE2 in varying doses) independent experiments. Percentages are based on the total number of oocytes examined.

Significant differences compared with the control: ^#^*P* < 0.001, ^*^*P* < 0.0001.
