# Supplementary material for: Involvement of the Serine Protease Inhibitor, SERPINE2, and the Urokinase Plasminogen Activator in Cumulus Expansion and Oocyte Maturation
Source: PLoS One. 2013 Aug 30;8(8):e74602. doi: 10.1371/journal.pone.0074602 (PMC3758271; doi:10.1371/journal.pone.0074602)
Supplement: Table S4 — Effects of PLAU protein on oocyte maturation. (DOC) [file pone.0074602.s011.doc]

**Table S4.** Effects of PLAU protein on oocyte maturation

| Oocyte stage | Control (%) | PLAU,  20 U (%) | Amiloride  300 μM (%) | SERPINE2+PLAU (%) | Amiloride+PLAU (%) |
| --- | --- | --- | --- | --- | --- |
| GV | 6.09 ± 2.43 | 3.53 ± 1.90 | 17.65 ± 4.72 | 4.55 ± 1.15 | 6.62 ± 3.13 |
| MI | 26.88 ± 4.52 | 10.96 ± 4.25 | 63.61 ± 10.24 | 38.97 ± 3.99 | 40.57 ± 4.76 |
| MII | 67.03 ± 6.48 | 85.51 ± 8.94^†^ | 18.73 ± 5.76^*^ | 56.48 ± 3.86 | 52.80 ± 4.87 |
| Number of COCs | 419 | 332 | 484 | 158 | 181 |

GV, germinal vesicle; MI, metaphase I; MII, metaphase II; COCs, cumulus–oocyte complexes.

Data are means ± SD of six independent experiments. Percentages are based on the total number of oocytes examined.

Significant differences compared with the control group: ^†^*P* < 0.05, ******P* < 0.0001.
